# Key features of an Hsp70 chaperone allosteric landscape revealed by ion-mobility native mass spectrometry and double electron-electron resonance

**DOI:** 10.1074/jbc.M116.770404

**Published:** 2017-04-20

**Authors:** Alex L. Lai, Eugenia M. Clerico, Mandy E. Blackburn, Nisha A. Patel, Carol V. Robinson, Peter P. Borbat, Jack H. Freed, Lila M. Gierasch

**Affiliations:** From the ‡ Department of Chemistry and Chemical Biology, Cornell University, Ithaca, New York 14853-2703,; the Departments of § Biochemistry and Molecular Biology and; ** Chemistry, University of Massachusetts, Amherst, Massachusetts 01003,; the ¶ School of Environmental, Physical, and Applied Sciences, University of Central Missouri, Warrensburg, Missouri 64093, and; the ‖ Physical and Theoretical Chemistry Laboratory, South Parks Road, Oxford OX1 3QZ, United Kingdom

**Keywords:** 70-kilodalton heat shock protein (Hsp70), allosteric regulation, chaperone DnaK (DnaK), conformational change, molecular chaperone, Hsp70, double electron-electron resonance, energy landscape, ion-mobility mass spectrometry

## Abstract

Proteins are dynamic entities that populate conformational ensembles, and most functions of proteins depend on their dynamic character. Allostery, in particular, relies on ligand-modulated shifts in these conformational ensembles. Hsp70s are allosteric molecular chaperones with conformational landscapes that involve large rearrangements of their two domains (*viz.* the nucleotide-binding domain and substrate-binding domain) in response to adenine nucleotides and substrates. However, it remains unclear how the Hsp70 conformational ensemble is populated at each point of the allosteric cycle and how ligands control these populations. We have mapped the conformational species present under different ligand-binding conditions throughout the allosteric cycle of the *Escherichia coli* Hsp70 DnaK by two complementary methods, ion-mobility mass spectrometry and double electron-electron resonance. Our results obtained under biologically relevant ligand-bound conditions confirm the current picture derived from NMR and crystallographic data of domain docking upon ATP binding and undocking in response to ADP and substrate. Additionally, we find that the helical lid of DnaK is a highly dynamic unit of the structure in all ligand-bound states. Importantly, we demonstrate that DnaK populates a partially docked state in the presence of ATP and substrate and that this state represents an energy minimum on the DnaK allosteric landscape. Because Hsp70s are emerging as potential drug targets for many diseases, fully mapping an allosteric landscape of a molecular chaperone like DnaK will facilitate the development of small molecules that modulate Hsp70 function via allosteric mechanisms.

## Introduction

Even in their native states proteins exist as conformational ensembles rather than as discrete, static structures. A protein function that strongly relies on the existence of conformational ensembles is allostery, defined as the influence of ligand binding at one site on a distant region of the protein. Allostery arises from ligand modulation of the populations of states on an energy landscape (1). High-resolution structures provide information about static states implicated in allostery but cannot shed light on the dynamic aspects of allosteric transitions or states that are transiently populated.

**Figure 1.**
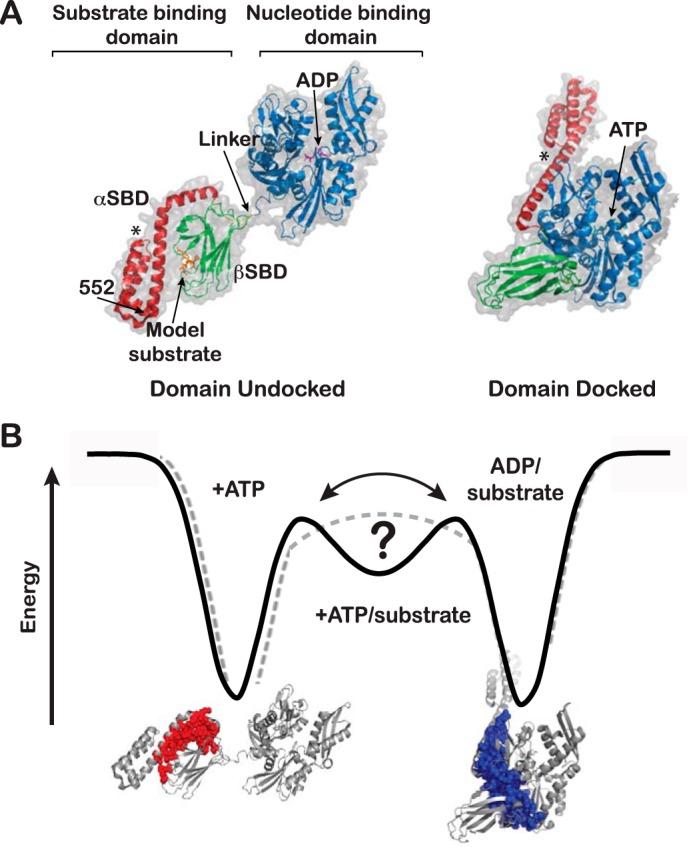
**Allosteric conformational rearrangement of DnaK.** *A*, *left*, domain-undocked DnaK (PDB entry 2KHO (6)). Bound ADP (*magenta*) was introduced into the structure using PDB entry 3ATU (54), and bound peptide substrate (*orange*) was introduced using PDB entry 1DKZ (5). *Right*, domain-docked DnaK (PDB entry 4B9Q (8)). ATP is shown in *cyan*. In both *panels*, the NBD is *blue*, the β-subdomain of the substrate-binding domain is *green*, the α-helical lid is *red*, and the conserved hydrophobic sequence of the interdomain linker (VLLL) is *yellow*. *, the unstructured C-terminal tail is not shown. (Structures were prepared using PyMOL (Schrödinger, LLC, New York).) *B*, simplified schematic of the interconversion between the domain-docked and domain-undocked states of DnaK in an energy landscape. The addition of both ATP and substrate ligands creates an intermediate state that may or may not be a true basin on the energy landscape. The structures shown illustrate the interaction surfaces that stabilize the two end-point allosteric states of DnaK. *Red surface*, βSBD/αSBD interface that forms in the domain-undocked structure; *blue surface*, SBD/NBD interface that forms in the domain-docked structure.

Hsp70 molecular chaperones are allosteric proteins that perform a multitude of chaperoning functions in the cell under normal physiological conditions and in response to stress (2, 3). Crucially, Hsp70 chaperones are paradigms of ligand-mediated modulation of energy landscapes, because their cellular functions rely absolutely on their allosteric transitions. Hsp70s consist of an N-terminal nucleotide-binding domain (NBD),^3^ which binds and hydrolyzes ATP (4), and a substrate-binding domain (SBD), which is made up of a β-sheet subdomain (βSBD) and an α-helical lid subdomain (αSBD; helices A–E) (5). A highly conserved linker connects the NBD and SBD, and the domains are followed by an unstructured C-terminal tail (Fig. 1*A*). All cellular functions of Hsp70s rely on a deceptively simple allosteric mechanism of substrate binding and release in which ATP binding leads to reduced substrate affinity (faster on/off rates). This mechanism, despite its apparent simplicity, is accompanied by a dramatic conformational change (Fig. 1*A*). Most insights into the structural basis of Hsp70 allostery are based on *in vitro* studies of the *Escherichia coli* Hsp70 DnaK and its component domains. In the ADP-bound state, the NBD and the SBD of DnaK behave like separate domains flexibly linked to one another by the interdomain linker (6, 7); the SBD has a high affinity for substrates, and the canonical binding site is “closed” by the interface formed between the βSBD and the αSBD (Fig. 1*A*). The relative orientations of the NBD and SBD in the ADP state have been shown to be restricted to a 30º cone based on paramagnetic relaxation enhancements and residual dipolar couplings (6). Upon exchange of ADP for ATP, the two domains of DnaK dock onto each other, and the interdomain linker forms an intimate part of the interdomain interface (8, 9). Concomitantly, the αSBD detaches from the βSBD, and the βSBD undergoes conformational rearrangements that lead to the opening of the substrate-binding site, thus favoring substrate release (Fig. 1*A*).

ATP binding to DnaK causes a decrease of 2 orders of magnitude in the affinity for substrates compared with the affinity in the presence of ADP. Conversely, substrate binding to the SBD stimulates the ATPase activity of the NBD (10, 11); therefore, at one point of the cycle, DnaK is bound to ATP and substrate at the same time. Indeed, NMR studies using an ATP hydrolysis-deficient mutant of DnaK (T199A) allowed the study of the ATP- and substrate-bound chaperone. Chemical-shift analysis revealed that substrate addition to the ATP-bound (domain-docked) DnaK drives the SBD toward its higher affinity conformation and favors interaction between the βSBD and the αSBD to close the binding site over the substrate. At the same time, the NBD/αLid interface is destabilized while the interdomain linker remains docked in what was termed the “allosterically active state” and proposed to be the state with high ATPase activity (11). In the ATP-bound state, domain docking is maintained by αSBD/NBD and βSBD/NBD interactions that involve the interdomain linker (*blue surface* in Fig. 1*B*), whereas in the ADP/substrate-bound state, the αSBD/βSBD interface is formed (*red surface* in Fig. 1*B*). Critical for chaperone function, the formation of these interfaces is mutually exclusive. Thus, in the allosterically active state, there is an energetic tug-of-war between the competing interfaces.

Several lines of research have shown that different members of the Hsp70 family populate many conformations along their allosteric cycles (12–17). In addition to the interdomain rearrangements that occur upon nucleotide binding and hydrolysis, the helical lid is dynamic and confers functional plasticity to Hsp70. For example, this plasticity allows Hsp70s to bind a variety of substrate conformations, from extended short peptides to near-native protein intermediate states (12, 14, 15).

Although past work has provided deep insights into the allosteric cycle of DnaK, there remain unanswered questions. How is the conformational ensemble populated at each point of the allosteric cycle, and how do ligands control these populations? Does ATP/substrate-bound DnaK exist as a discrete, partially docked intermediate as part of the energy landscape, or does it result from the rapid interconversion between the domain-docked and -undocked states (Fig. 1*B*)? Based on our earlier NMR results (11), the allosteric state is formed in the presence of substrate and ATP. Some of its structural features resemble the domain-undocked state of DnaK (like the interface between the α-helical lid and the βSBD), whereas others resemble the domain-docked state, such as the NBD-associated interdomain linker. Thus, very sensitive methods are needed to distinguish the possibilities that the allosteric intermediate is a discrete state or results from interconversion of two states. The present study deploys two complementary techniques, ion-mobility native mass spectrometry (IMMS) and double electron-electron resonance (DEER), to answer these questions and elucidate the conformational ensemble of DnaK under biologically relevant ligand-bound conditions (ATP, ADP/substrate, and ATP/substrate). IMMS and DEER are performed on proteins in the gas phase and in frozen samples, respectively; thus, individual conformers present in the ensembles are not expected to interconvert. Both techniques have been successfully employed in the past to characterize the layout of conformational ensembles as well as the dynamics of individual conformers (18–21). Here, IMMS and DEER have allowed us to survey the arrangement of the conformational ensembles of DnaK under different ligand-bound conditions and to identify sparsely populated conformers. Moreover, these approaches establish that ATP/substrate-bound DnaK populates a discrete partially docked state.

## Results

### Experimental design

To explore the nature and dynamics of the conformational ensemble of DnaK in different ligand conditions, we selected two methods that yield descriptions of multiple co-existing states: IMMS and DEER. In all experiments, we used a DnaK mutant carrying the mutation T199A, which impairs ATP hydrolysis (7, 22, 23) and thus allows the study of the protein stably binding ATP and ATP/substrate (11). In this work, “DnaK” refers to T199A DnaK.

IMMS measures the mobility of the protein ions in the gas phase through a helium-filled tube (drift tube) under a weak electric field. While the drift tube separates the ions in the gas phase based on their mobility (measured as “arrival time”), the MS dimension measures the masses of the separated ions. Thus, ions with the same mass and the same charge but different “conformation” will have different arrival times. The two-dimensional separation of ions allows precise measurement of each ion's arrival times from a complex mixture of species. The rotationally averaged collision cross-section (CCS, in Å^2^) of a given species is calculated from its arrival time. CCS in turn depends on the ion's mass, shape, and charge (24) and therefore yields insight into the nature of the particular species observed. Using the projection approximation method (25, 26), we estimated that the docked and undocked states of DnaK would have a difference in CCSs large enough to be separated by IMMS. (Note that because the constructs of DnaK analyzed by crystallography and NMR lack the C-terminal domain and part of the αSBD (Fig. 1*A*), the projection approximation was applied only to evaluate the differences on CCS between the docked and undocked states.) Indeed, the calculated CCSs were significantly different: 4193 Å^2^ for the ATP-bound (from PDB entry 4B9Q (8)) and 4681 Å^2^ for the ADP/substrate-bound (from PDB entry 2KHO (6)). Thus, we concluded that we could detect the different DnaK conformers in the ensemble by IMMS. (Note that for the substrate-bound SBD, the presence of the peptide in the binding groove did not change the CCS estimated for the SBD without substrate (not shown)).

**Figure 2.**
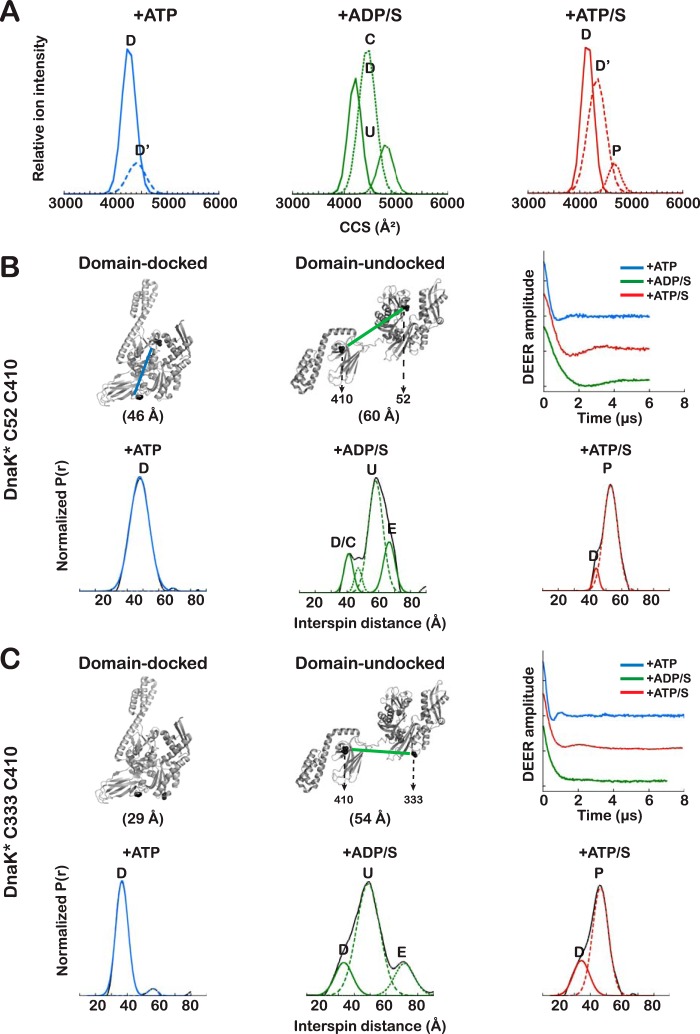
**The conformational ensembles of DnaK deduced from IMMS and DEER measurements.** *A*, CCS distributions are shown for all ligand-bound forms of DnaK for ion +17, because this ion is populated under all ligand-bound conditions (ATDs for some ions are shown in supplemental Fig. S1). ATDs for all ions were fit to Gaussian components, as described under “Experimental procedures,” and for each component, the CCS was calculated and used to convert the *x* axis to CCS (Å^2^). The components of the same ion are indicated as docked (*D*), domain-docked with detached α-helical lid (*D*′), domain-undocked with domains close (*C*), undocked (*U*), or partially docked (*P*). *B* and *C*, *left*, estimated distance between spins in DnaK* Cys^52^-Cys^410^ and DnaK* Cys^333^-Cys^410^, respectively, based on Cβ–Cβ distance in PDB 2KHO and 4B9Q. The estimated distances do not consider the spin-label side chains. *Right*, baseline-subtracted and normalized data showing time evolution of the interspin coupling. *Bottom panels*, interspin distance distributions for spin labels on DnaK* in the indicated ligand-bound states. *Solid lines*, experimental data; *dotted lines*, fitted Gaussian components. *Blue*, ATP-bound; *green*, ADP/substrate-bound; *red*, ATP/substrate-bound.

**Figure 3.**
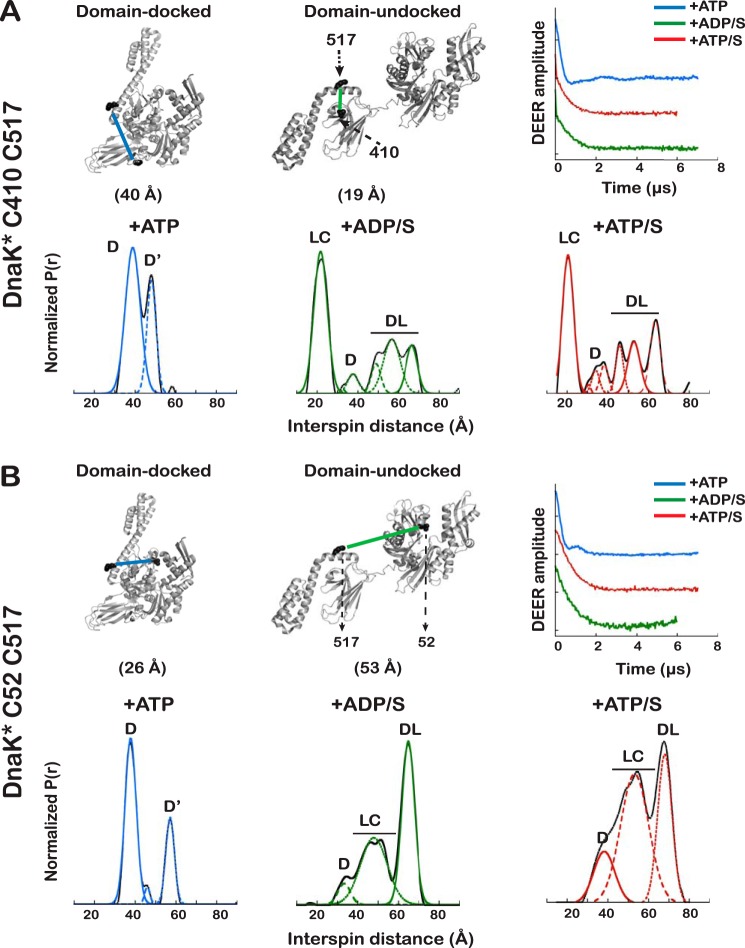
DEER-derived distance distributions in DnaK* Cys mutants that report on dynamics of the α-helical lid. *A* and *B*, *left*, measured distance between spins in DnaK* Cys^410^-Cys^517^ and DnaK* Cys^52^-Cys^517^, respectively, based on Cβ–Cβ distance in PDB entries 2KHO and 4B9Q. The estimated distances do not consider spin-label conformers. *Right*, baseline-subtracted and normalized data showing time evolution of the interspin coupling. *Bottom panels*, interspin distance distributions for spin labels on DnaK* in the indicated ligand-bound states; *solid lines*, experimental data; *dotted lines*, fitted Gaussian components. *Blue*, ATP-bound; *green*, ADP/substrate-bound; *red*, ATP/substrate-bound. *D*, docked; *U*, undocked; *DL*, dynamic lid; *LC*, lid closed. Because of the higher uncertainty due to signal heterogeneity and dynamics, the distributions at mid-distances for the Cys^52^-Cys^517^ mutant were fit to only one Gaussian (three total components).

DEER yields the distance distributions between two spin systems for a frozen sample and is sensitive to distances between 20 and 80 Å (27). Spins are introduced by attaching *S*-(1-oxyl-2,2,5,5-tetramethyl-2,5-dihydro-1H-pyrrol-3-yl)methyl methanesulfonothioate (MTSL) to Cys pairs engineered into the protein at strategic and tolerant locations (28, 29). The populations of the contributing conformational states can be quantitatively fit to the experimentally observed distance distributions (30). Cys residues were introduced into DnaK* (where the asterisk indicates a truncated version of DnaK that lacks the C-terminal 89 residues but retains full allosteric function (11, 31); see “Experimental Procedures” for descriptions of constructs) at positions that were predicted from known structures to yield interspin distances that are expected to undergo large changes, depending on the ligand bound (Figs. 2 (*B* and *C*) and 3). Based on established nucleotide-induced shifts in Trp fluorescence emission spectra (32), all double Cys mutants studied retain the ability to undergo the conformational changes associated with ATP binding to full-length DnaK (supplemental Fig. S5*A*). Two types of Cys pairs were selected; the Cys^333^-Cys^410^ and Cys^52^-Cys^410^ pairs report on interdomain, βSBD/NBD docking, whereas the Cys^52^-Cys^517^ and Cys^410^-Cys^517^ pairs report on movements of the α-helical lid with respect to the NBD and the βSBD, respectively.

### The conformational ensemble of DnaK in the presence of ATP: the NBD and βSBD are tightly docked, and the α-helical lid populates both NBD-associated and dissociated states

Both IMMS and DEER results are consistent with a conformational ensemble for DnaK+ATP that is relatively homogeneous and populated by compact species. The arrival time distributions (ATDs) in IMMS of ATP-bound DnaK show one principal peak that is narrow, consistent with an ensemble composed of a well-defined species (supplemental Fig. S1*A*). This peak (CCS = 4218 ± 28 Å^2^) is assigned to the NBD-SBD docked state (D). Fitting the ATDs of each ion in the MS spectrum to Gaussian components (see supplemental Fig. S1 for fitting criteria) reveals the presence of an additional, significantly less populated state with an arrival time longer than that of the principal (domain-docked) species (CCS = 4423 ± 23 Å^2^) (Fig. 2*A*, Table 1, and supplemental Fig. S1*A*); the combined use of IMMS and DEER results (described in detail below) allows us to assign this minor component to an alternate domain-docked state (D′). Consistent with the presence of ATP-bound structures that do not fluctuate significantly (33), CCS distributions derived from the observed ATDs span a narrow range (190 Å^2^; see supplemental Fig. S4).

**Table 1:** **Calculated CCS (in Å^2^) for each component of the conformational ensembles of different ligand-bound DnaK (ion +17)** Values represent the average of three independent measurements ± S.D. The individual values are plotted in supplemental Fig. S3.

Ligand	C1	C2	C3
ATP	4218 ± 28	4423 ± 23	
ADP/S	4221 ± 16	4475 ± 35	4815 ± 21
ATP/S	4182 ± 47	4335 ± 29	4649 ± 42

DEER measurements enable us to interrogate the conformational ensembles in terms of intramolecular distance distributions and thus to refine the structural interpretations of the ensembles. For example, the DEER-derived distance profiles of DnaK* carrying spin labels at Cys^333^ and Cys^410^ and at Cys^52^ and Cys^410^ report on interdomain docking (Fig. 2 (*B* and *C*) and Table 2). For both variants, the distance distributions show a single peak around a distance; for the Cys^52^-Cys^410^, the measured distance agrees with the high-resolution crystal structure models (see Refs. 8 and 9, Fig. 2 (*B* and *C*, *left panels*), and Table 2). It is noteworthy that the interspin distance between Cys^333^ and Cys^410^ in the ATP-bound state reported by DEER is significantly larger than the distance measured from either reported crystal structure (37 *versus* 29 Å, respectively (8, 9)). This discrepancy could arise from either the orientation of the spin labels or true differences between the structures in solution and in the crystal. The presence of only one distribution in the interspin distance profile supports the conclusion that the interface between the NBD and βSBD is stable when DnaK is bound to ATP. Thus, the ensemble populates a single predominant conformation assigned to the NBD/SBD-docked state (D).

**Table 2:** Parameters for the individual Gaussian components of the DEER distance distributions for spin-labeled DnaK*

DnaK*	Ligand	Maximum*^a^*	Width*^b^*	Area*^c^*
		Å	Å	%
Cys^52^-Cys^410^	ATP	42	9	100
	ATP/substrate	44	4	10
		53	9	90
	ADP/substrate	44	7	12
		59	10	77
		67	6	11
Cys^333^-Cys^410^	ATP	37	6	100
	ATP/substrate	34	10	24
		46	10	76
	ADP/substrate	33	9	14
		49	15	74
		72	11	12
Cys^410^-Cys^517^	ATP	39	7	67
		48	4	33
	ATP/substrate	21	5	40
		36	8	14
		56	3	8
		53	7	18
		64	5	20
	ADP/substrate	22	7	48
		37	6	6
		49	4	5
		57	10	28
		67	5	13
Cys^52^-Cys^517^	ATP	38	5	71
		57	4	29
	ATP/substrate	38	10	16
		53	13	53
		68	7	31
	ADP/substrate	33	6	7
		48	13	43
		65	6	50

*^a^* Position of the center of the peak.

*^b^* Width of the peak at 50% height.

*^c^* Percentage of the area under the curve for the component divided for the total area of the distribution.

However, when a spin label is placed on a Cys introduced in the α-helical lid of DnaK* (either in the Cys^410^-Cys^517^ or the Cys^52^-Cys^517^ variant), two discrete distributions of the interspin distances can be clearly distinguished (Fig. 3, *A* and *B*). Because DnaK* Cys^333^-Cys^410^ and DnaK* Cys^52^-Cys^410^ reported that the NBD and βSBD are docked at all times, we assign the peaks in the DEER-derived distance distribution for spin label pairs with one site on the α-helical lid to a docked conformation with the helix stably associated with the NBD (D) and a docked conformation where the α-helical lid is dissociated from the NBD (D′) (Table 2). This result from DEER is consistent with the conclusion from IMMS that there is a second species in the ensemble of ATP-bound DnaK. This D′ state is not the result of introducing the Cys^517^ mutation, because the effect of domain docking on the fluorescence of Trp^102^ (sensitive to the position of the lid with respect to the NBD) is the same for the Cys mutants and for the non-mutated version (supplemental Fig. S5*A*).

Putting together the results from IMMS and DEER analysis of ATP-bound DnaK has provided a consistent picture; in this state, the NBD and βSBD are always docked, but for a significant fraction of the population (∼30%), the α-helical lid is detached from the NBD (Table 2).

### The conformational ensemble of DnaK in the presence of ADP and substrate: DnaK populates many states, the NBD and βSBD are largely undocked, and the α-helical lid is dynamic

Previous work has established that ADP- and ADP/substrate-bound Hsp70s adopt more than one conformation, that the two domains can move with respect to each other, and that the α-helical lid is dynamic (6, 7, 11, 13–17). Here, IMMS and DEER results offer a defined understanding of the heterogeneity of the conformational distribution. The ATDs derived from IMMS for ADP/substrate-bound DnaK are wider than those for DnaK in other nucleotide/substrate conditions (Fig. 2*A* and supplemental Fig. S1*B*). Moreover, consistent with ADP/substrate-bound DnaK sampling of a spectrum of related conformations (33), in the presence of ADP and the model peptide substrate p5 (CLLLSAPRR) (7, 34, 35), all ions observed by IMMS populate states that span a large range of CCS (730 Å^2^) (supplemental Fig. S4).

The CCS distributions of ADP/substrate-bound DnaK show that in this state, the protein visits at least three discrete conformations (the ion with 17 charges was selected to compare all ligand-bound DnaK states because it is populated under all conditions (Fig. 2*A*, supplemental Figs. S1 and S2, and Table 1)). We assigned the fully domain-undocked state U to the peak with CCS = 4649 Å^2^, the largest species populated by DnaK; an additional state C (CCS = 4335 Å^2^) was assigned to a domain-undocked form where the NBD and SBD are largely independent of each other but spend time at a smaller interdomain distance, and a state with a smaller CCS (4182 Å^2^) was assigned to the docked state, which is the most compact structure visited by DnaK in all tested conditions. Previous NMR experiments did not detect this compact state of DnaK when ADP and substrate were bound (7, 11). This highly populated state may arise from the presence in solution of a previously undetected docked form of ADP/substrate-bound DnaK. Alternatively, and we believe more probably, this state can be attributed to the collapse of the undocked DnaK in the gas phase, because collapse in the gas phase has been reported previously for some proteins (33, 36, 37). We cannot rule out the presence of a small population of the domain-docked state in solution for ADP/substrate-bound DnaK (also detected for the mitochondrial Hsp70 in the presence of ADP (17)), but this species seems to be overrepresented in the ensemble observed by IMMS. We propose that this gas phase-compacted state is indistinguishable from domain-docked DnaK and arises from collapse during the ionization process. Interdomain distances determined by DEER (see below) support the conclusion that an NBD/SBD docked state exists as part of the ADP/substrate-bound DnaK ensemble.

ADP/substrate-bound DnaK* is conformationally heterogeneous, as seen by DEER (Figs. 2 (*B* and *C*) and 3 and Table 2). For the Cys^52^-Cys^410^ spin pair in the presence of ADP/substrate (using the well-characterized peptide NR (NRLLLTG) as a model substrate (5, 11)), the most highly populated species has a distance distribution centered at 59 Å; we assign this species to the domain-undocked form (U). The distance distribution for this state is broad, as expected, given that the two domains can move with respect to one another and can populate many closely related conformations (6, 7, 11). In this ensemble, DEER results show that DnaK* can also visit a distinct conformation with a shorter interdomain distance (44 Å) that we assign to the domain-docked conformation. This docked state represents only ∼10% of the population (Table 2) and thus could be present in the solution conformational ensemble and not have been observed by NMR. This NBD/βSBD docked conformation that was detected here by both IMMS and DEER resembles a docked form observed for the ADP-bound state by single molecule Förster resonance energy transfer (FRET) (16). A third component was observed in the DnaK* Cys^52^-Cys^410^ ensemble; this component fits to an interspin distance of 65 Å, longer than the maximum distance expected for the undocked form (54 Å). Because DEER distance distributions have greater uncertainty as distances increase (see discussion in the supplemental data and Refs. 28 and 38), we cannot determine the interspin distances precisely. Nonetheless, this species is only observed when DnaK* is bound to both ADP and substrate and probably reports on a more extended configuration of the linker. The same populated states D, U, and E can be identified in the distance profiles derived from DnaK* with spin labels at Cys^333^ and Cys^410^ (Fig. 2*C* and Table 2).

The distance profiles measured by DEER when a spin-label is placed on Cys^517^ in the α-helical lid reveal that the lid is dynamic and moves away from the βSBD, even when substrate is tightly bound (Fig. 3). The interspin distance profile for DnaK* with spin labels at Cys^410^ and Cys^517^ shows that the predominant species in the presence of ADP/S has the “lid closed” over the substrate (LC, centered at 22 Å). Cys^517^ moves away from the substrate-binding site, populating the D state and a family of states with interspin distances between 37 and 66 Å (*DL* in Fig. 3), reflecting the dynamics of the lid detaching from the βSBD. This behavior of the lid was also seen in DEER data for DnaK* Cys^52^-Cys^517^ (Fig. 3*B* and Table 2).

### In the presence of ATP and substrate, DnaK populates a partially docked state

A major question motivating this study was whether the conformational ensemble of DnaK bound to ATP and substrate arises from a discrete, partially docked intermediate state or from dynamic sampling of domain-docked and -undocked forms. Our complementary approach using IMMS and DEER has unequivocally answered this question, showing the existence of a discrete allosterically active state of DnaK, different from the docked and undocked forms. We further observe that dynamics of the helical lid subdomain lead to a set of conformational substates for the allosterically active state. First, DEER results on the two variants of DnaK* with complementary pairs of spin-labeled Cys residues establish the existence of a state populated by ATP/substrate-bound DnaK that possesses a stable NBD-βSBD interface, whereas the α-helical lid is either closed over the substrate or, in part of the population, detached from the substrate binding site. In the presence of ATP and substrate, DnaK* Cys^52^-Cys^410^ populates a state P, with distance distribution centered at 53 Å, significantly shorter than that observed for the U state (Fig. 2*B* and Table 2) and longer than that observed for the ATP-bound docked state. This peak represents 90% of the population and reflects a discrete state different from the U and the D states. The absence of a peak corresponding to the U state argues that the interdomain interface, in which the interdomain linker is directly participating in the presence of ATP, is stable. The ensemble observed for ATP/substrate-bound DnaK* Cys^52^-Cys^410^ also contains a more compact conformation (D, centered at 44 Å). The distance distribution profile of DnaK* Cys^333^-Cys^410^ (Fig. 3*A* and Table 2) also shows a distance that corresponds to the domain-docked state (34 Å) and a distance shorter than that corresponding to the completely undocked state (46 Å).

The distance profile of the DnaK* Cys^410^-Cys^517^ pair (21 Å) (Fig. 3*A*) reveals that in the predominant population of this partially docked state of ATP/substrate-bound DnaK*, the α-helical lid is closed over the substrate (LC). As observed for the domain-undocked structure, we found in the distance distribution a family of structures that report on the dynamics of the helical lid (DL). This behavior of the lid was also observed in DnaK* Cys^52^-Cys^517^ (Fig. 3*B*).

The partially docked state can also be observed by IMMS. The position of the ion peaks on the m/*z* axis in a native mass spectrum is primarily determined by the protein solvent-accessible surfaces, where a larger solvent-accessible surface can accommodate more charges during the ionization process (39, 40). For DnaK, distributions of the ions generated in different ligand conditions shift on the *m*/z axis (see example in supplemental Fig. S2); for example, the ion distribution of ADP/substrate-bound DnaK is centered at +18, whereas that for ATP-bound DnaK is centered at +16 and that for the ATP/substrate-bound state is generally centered at +17. These results reflect the size of species in the respective conformational ensembles; that for ADP/substrate-bound DnaK contains the largest species, whereas that for ATP-bound DnaK contains the most compact species, and the size of species in the conformational ensemble in the presence of ATP and substrate is between that of the ATP- and ADP/substrate-bound states.

The separation of the DnaK species in the ion-mobility dimension also reveals a partially docked state in the presence of ATP and substrate (Fig. 2*A*, Table 1, and supplemental Figs. S1*B*–S4). The CCS distributions of the species show that in addition to the domain-docked states D and D′ (4182 ± 47 and 4335 ± 29 Å^2^, respectively), there is a state where ATP and substrate are both bound to DnaK with a CCS distinct from that of the D or U states (4649 ± 42 Å^2^), whereas the domain-undocked (U) species is not populated at all (Fig. 2*A* and Table 1). We assigned this state to the partially docked form (P), also defined as the allosterically active state. We observed that in the presence of ATP and substrate, the CCS values of all ions are fit to Gaussian components that span a range of values intermediate between those of the docked and undocked forms (532 Å^2^; supplemental Fig. S4), arguing for dynamic behavior of the predominant state that is intermediate between the docked and undocked forms (33).

### The equilibrium distribution between the fully docked, undocked, or partially docked allosterically active states can be shifted by ligands or mutations

**Figure 4.**
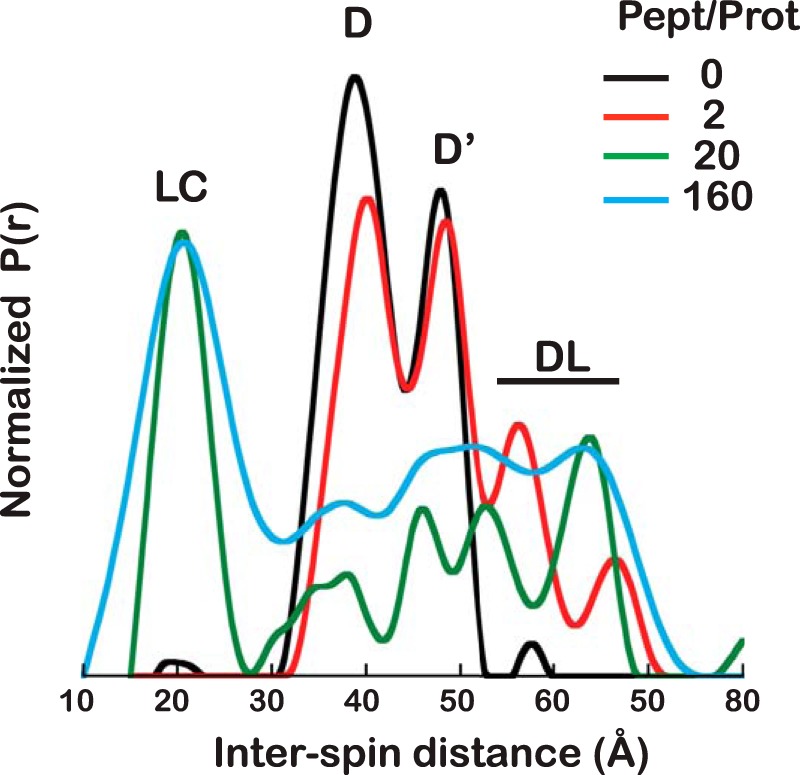
Titration of ATP-bound DnaK* Cys^410^-Cys^517^ with NR peptide. Distance distributions of DnaK* Cys^410^-Cys^517^ in the presence of ATP as NR peptide is added in increasing concentrations, at peptide/protein ratios of 0, 2, 20, and 160 (*black*, *red*, *green*, and *blue curves*, respectively). *D*, docked; *D*′, domain docked with detached α-helical lid; *DL*, dynamic lid; *LC*, lid closed.

Fig. 4 shows the changes in the DEER-derived distance distribution of ATP-bound DnaK* Cys^410^-Cys^517^ as substrate is added. In the absence of substrate, ATP-bound DnaK* shows two peaks corresponding to the domain-docked state, one arising from the α-helical lid-associated state and the other from the α-helical lid-dissociated state (D and D′). After a 2-fold molar excess of peptide is added to the protein, a family of states at longer interspin distances is seen that appears to correspond to states where the NBD and βSBD remain docked but the helical lid becomes more dynamic (Fig. 4, *red curve*). When a 20-fold molar excess of peptide is added, the partially docked state with the lid closed over the substrate (LC) is populated at the expense of the domain-docked species and is in equilibrium with the “dynamic-lid” species (Fig. 4, *green curve*); equilibrium is reached upon the addition of a 160-fold molar excess of peptide (*blue curve* in Fig. 4).

**Figure 5.**
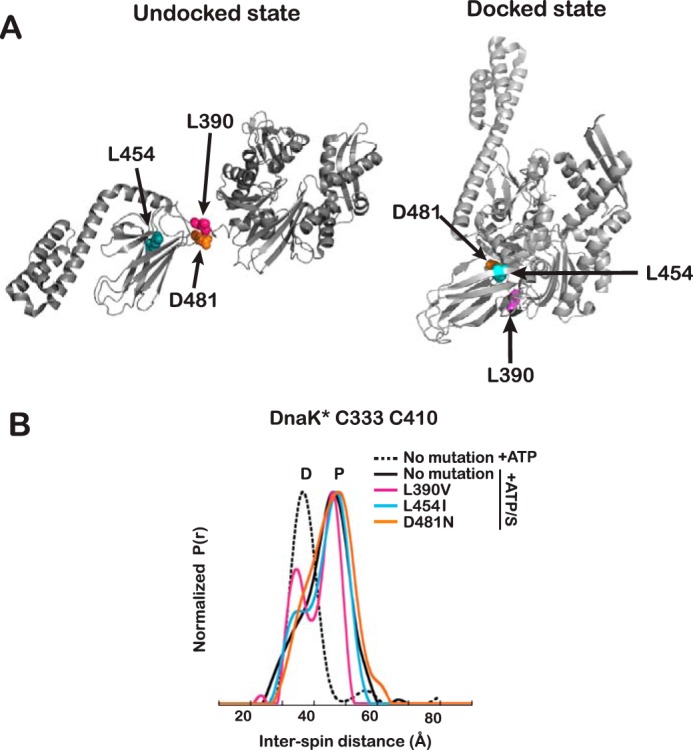
**Impact of mutations that modulate the stability of the interdomain interface.** *A*, mutated residues represented as *spheres* in the structures of DnaK in the undocked (PDB entry 2KHO) and docked (PDB entry 4B9Q) states. *B*, interspin distance distributions for spin labels on ATP/substrate-bound DnaK* Cys^333^-Cys^410^ carrying mutations that stabilize (L390V and L454I) or destabilize (D480N) the interdomain interface. Labeling of the peaks is as in Fig. 3. Data are shown for DnaK* Cys^333^-Cys^410^ without the interface mutations for comparison (*black lines*). Distance distributions for each mutant are shown in *magenta* for L390V, *cyan* for L454I, and *orange* for D480N.

The allosterically active state is the result of an energetic tug of war between competing orthogonal interfaces: the NBD/βSBD and NBD/αSBD interfaces (favored by the presence of ATP) and the αSBD/βSBD interface (optimally formed in the presence of substrate) (11). Because DEER allows us to directly observe features on the conformational landscape of DnaK, we explored by DEER the impact of several mutations previously characterized by NMR, L390V, L454I, and D481N (11), using the spin-labeled DnaK* Cys^333^-Cys^410^ variant (Fig. 5). L390V and L454I are known to stabilize the interdomain interface, whereas D481N weakens it (these mild mutations did not perturb the structural features of the protein (supplemental Fig. S5*C*)). Fig. 5 shows the distance distributions of DnaK* Cys^333^-Cys^410^ (+ATP/substrate) for each mutant, compared with the distance distributions of the DnaK* double Cys variant without the mutation (in the presence of both ATP and ATP/substrate); the degree of docking was quantified by measuring the docked component in the curves and the percentage reported in Table 3. When observing the NBD/βSBD distance between Cys^333^ and Cys^410^ in L390V and L454I, the stabilization of the interface is reflected in the distance profiles, because the docked state is more highly populated than in the non-mutated DnaK* (Fig. 5). This interface-stabilizing effect is more marked for the L390V mutation and subtler for the L454I variant. Conversely, the D481N mutation causes a destabilization of the NBD/SBD interface (11).

**Table 3:** Effect of interdomain interface mutations on ATP/substrate-bound DnaK* Cys^333^-Cys^410^ as measured by DEER

DnaK* Cys^333^-Cys^410^	Percentage undocked*^a^*	Effect on NBD/SBD interface
No mutation	76	
L390V	60	Stabilization
L454I	68	Stabilization
D481N	82	Destabilization

*^a^* Percentages were calculated as the ratio of the area of peaks identified as undocked conformers divided by the total area of all peaks, based on the curves shown in Fig. 5.

## Discussion

**Figure 6.**
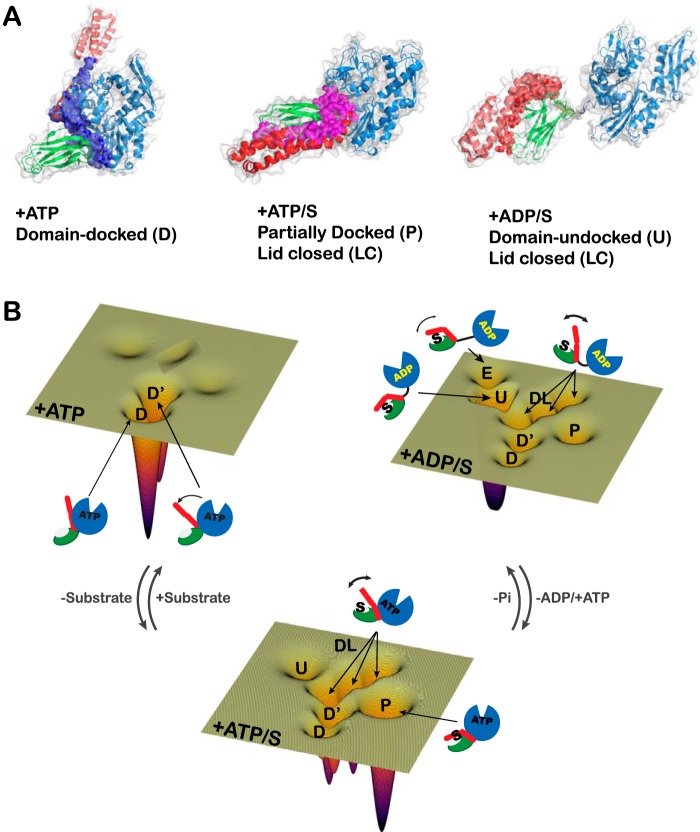
*A*, schematic representation of the allosterically active state. Shown is a model of the allosterically active state, where the *pink spheres* represent the interdomain interface formed between NBD and the linker, and between the α and β subdomains, when ATP and substrate are bound. The backbone of DnaK has been *colored* as in Fig. 1*A. B*, schematic representation of the allosteric energy landscape of DnaK under different ligand-bound conditions. The conformations of DnaK under all nucleotide and substrate conditions detected by IMMS and DEER are represented by *schematics* on an energy landscape. Each energy landscape contains all of the conformations present in the ensemble that were detected by DEER and IMMS. The depth of each conformational energy well is proportional to the relative abundance based on the DEER measurements.

The present work deployed two powerful methods, IMMS and DEER, to reveal detailed features of the allosteric landscape of the bacterial Hsp70, DnaK (Fig. 6). This characterization elucidated how the chaperone's conformational ensembles shift in response to different ligands (ATP, ADP/substrate, and ATP/substrate) and determined that the allosterically active state of DnaK (ATP/substrate-bound) is a partially docked discrete intermediate.

IMMS reports on the average collision cross-section of all of the DnaK species in the presence of particular ligands, with the advantage of detecting ions without labeling. Ions are detected in the gas phase so that minimal alteration of the conformational ensemble present in solution is anticipated to happen after the ionization process (41). DEER complements IMMS by measuring the distance distributions between two spin labels specifically engineered to monitor conformational changes in DnaK upon ligand binding. Like IMMS, these measurements give a snapshot of the conformational ensembles present in solution under a given set of ligand conditions, because DEER spectra are obtained at 60 K on quick-frozen samples, such that the protein does not undergo conformational transitions before the distance distributions are measured.

In the presence of ATP, both IMMS and DEER find one predominant conformation of DnaK in which the NBD and βSBD are docked and the molecule is compact (Fig. 2). The predominance of the docked state argues that the NBD/βSBD interface is stable. Interestingly, both methods reveal that there is another component of the population (state D′ in Figs. 2 and 3) wherein the NBD and the βSBD are tightly docked but the α-helical lid detaches from the NBD. The helical lid has been previously shown to be dynamic in the ADP-bound form of Hsp70s (12, 13, 16, 17) and in the docked state in the presence of AMP-PNP (12, 13), but dynamics of the lid had not been seen previously in ATP-bound Hsp70s. Note that previous descriptions of the ATP-bound state of DnaK based on crystallography gave the impression of a stable association of the helical lid with the NBD. But in one case, it was necessary to immobilize the α-helical lid onto the NBD through the introduction of a disulfide bridge between residues 529 and 47 to make ATP-bound DnaK amenable to crystallization (8). In the other crystal structure of ATP-bound DnaK (9), several mutations were made in the loops of the SBD to successfully crystallize this state, and these mutations pushed the construct toward a stable association of the lid with the NBD, as had been seen for Hsp110 (42). In summary, the ensemble of ATP-bound DnaK stably populates states with the NBD/βSBD docked, and there is a distribution between helical lid-associated and -detached states in equilibrium with each other.

The observation of a form of DnaK with ATP bound and the NBD and βSBD docked, but with the α-helical lid detached from the NBD, suggests a mechanism for the conformational changes induced by the addition of substrate (Fig. 4); upon substrate binding, the lid-detached form (D′) shifts into conformations where the lid becomes more dynamic (DL), which favors the allosterically active state, in which the SBD adopts a conformation more like the domain-dissociated form, but the interdomain linker remains docked to the NBD, and the interdomain distance is shorter (a more compact state than ADP-bound DnaK).

In the presence of ADP and substrate, results from both IMMS and DEER are consistent with the existence of a dynamic, heterogeneous population of states, as has been described for members of the Hsp70 family (6, 7, 11–13, 16, 17). For example, interdomain distance measurements by single-molecule FRET on mtHsp70 are consistent with the bulk of the population sampling a state in which the two domains are dissociated and show relative motion (16), as deduced previously for DnaK from NMR (6, 7, 11). Within this ensemble, we also detected a small population that is more compact (overrepresented by IMMS), which was not detected by the NMR techniques used previously to study DnaK (6, 7, 11), and presumably reflects the domain-docked state. The population of this state as estimated from DEER results is ∼10%, which is probably below the detection limits of earlier NMR studies. Additionally, DEER reports on a set of conformations that are more extended than the main population (E state in Fig. 2, *B* and *C*) and are only populated by 12% in the presence of ADP/substrate. The interspin distance is too long to be reliably determined (∼72 Å), but the novel conclusion that there exists a state with the domains more substantially extended is valid.

As expected, the α-helical lid is closed over the βSBD in the presence of ADP/substrate and ATP/substrate (Fig. 3), but in a large fraction of the population, this lid is dynamic (DL states in Fig. 3) and moves away from the substrate-binding site, populating a family of related structures. These observations are consistent with several previous reports (using single-molecule FRET (12, 13, 16, 17), electron paramagnetic resonance (15), and atomic force microscopy (14)) that described the α-helical domain of many Hsp70s as dynamic and correlated the dynamics with the binding of large or partially folded substrates.

We sought to determine in this work whether the allosterically active (ATP/substrate-bound) state of DnaK deduced by NMR (11) was an entity populated in the ensemble. The results from both IMMS and DEER, which offer faster time resolution than NMR, enabled us to conclude that the allosterically active state is a discrete, partially docked entity. As in the ATP-bound state, the presence of ATP bound to the NBD stabilizes the NBD/interdomain linker interface, and the molecule does not significantly populate the undocked state. From the DEER distance distributions, we conclude that the α-helical lid is dynamic in the allosterically active state. Based on the distances distribution and considering previous NMR data (11), we constructed a “model” structure incorporating the main features of the most represented structure in the landscape of ATP/substrate-bound DnaK (Fig. 6*A*); the β-subdomain is in proximity with the NBD, the interdomain linker is docked into the NBD, and the helical lid is closed over the bound substrate. This structure is consistent with the DEER-measured interspin distances, except that the measured distance between Cys^333^ and Cys^410^ is longer than in the model structure (and in the structure of ATP-bound DnaK). Given the consistency of distances from other labeling sites, we speculate that this site in the protein is in a dynamic portion of the backbone and that the spin label at this site may favor one preferred orientation.

The conformational landscape of an allosteric protein is encoded by its amino acid sequence (43). Using the powerful IMMS and DEER methods, we were able to see how the allosteric landscape of DnaK was perturbed by mutations that have only a small effect on local structure but map to a key interdomain interface that contributes to the allosteric balance (Fig. 5). Indeed, these mutations were known to cause stabilization (L390V and L454I) or destabilization (D481N) of the NBD/βSBD interface in the ATP-bound states (11). In the presence of ATP and substrate, these mutations specifically act on the modulation of the NBD/βSBD interface, altering the equilibrium among the species in the population and stabilizing or destabilizing the interface.

Based on the populations of states sampled by DnaK under all physiologically relevant ligand conditions, we conclude that the allosteric cycle of DnaK relies heavily on dynamic sampling and conformational population shifts on an energy landscape (1, 44) (Fig. 6*B*). This case is particularly interesting because it is the balance of the energy of the interfaces (which depend on the ligand bound) that adapts the conformational landscape to optimize the chaperone function.

Because Hsp70s are emerging as potential drug targets for many diseases, understanding the conformational ensemble at each point of their allosteric cycle gives us opportunities for selective intervention and modulation of Hsp70 activities.

## Experimental procedures

### Strains and proteins

All DnaK variants used here carry the mutation T199A to reduce their ATPase activity and allow observation of ATP-bound complexes. In this work “DnaK” always refers to T199A DnaK. All DnaK variants were generated by site-directed mutagenesis from the DnaK gene cloned into pMSK (7, 34), and all clones were fully sequenced. DEER analysis was carried out on the C-terminally truncated construct DnaK(1–552)YE (referred to as DnaK*), which lacks the helical bundle of the α-helical lid and has a less stable secondary structure in helix B (31), leading to a weaker βSBD/α-helical lid interaction. In the absence of substrate, the truncation does not affect the ATP-bound state (revealed by the similar basal ATPase activities of the full-length DnaK and DnaK(1–552) (31)), but the truncated variant displays a reduction in the degree to which the substrate shifts the equilibrium toward the domain-undocked ensemble (11). In addition to the truncation at residue 552 to remove sequences that render it aggregation-prone (Fig. 1*A* and supplemental Fig. S4*C*), DnaK* carries a Leu-Leu to Tyr-Glu mutation (YE) at residues 542–543 to avoid binding of the end of the terminal helix into the substrate-binding site (7). DnaK variants studied include DnaK, DnaK Cys^410^-Cys^517^, DnaK (1–552)YE (DnaK*), DnaK* Cys^333^-Cys^410^, DnaK* Cys^52^-Cys^410^, DnaK* Cys^410^-Cys^517^, DnaK* Cys^52^-Cys^517^, DnaK* Cys^333^-Cys^410^ L390V, DnaK* Cys^333^-Cys^410^ L454I, DnaK* Cys^333^-Cys^410^ D481N, DnaK* Cys^410^-Cys^517^ L390V, DnaK* Cys^410^-Cys^517^ L454I, and DnaK* Cys^410^-Cys^517^ D481N. Proteins were overexpressed in *E. coli* BB1553 (BBMC4100 Δ*dnak52*::Cam^R^, *sidB1*) (45) and purified by anion-exchange and ATP-affinity chromatography as described (34). For the Cys-containing mutants, 1 mm DTT was kept in all buffers until labeling.

After purification, the non-Cys-containing proteins were unfolded in 6 m urea buffer (10 mm potassium phosphate, 5 mm DTT, and 6 m urea, pH 7.5), concentrated using Amicon centrifuge filters (Millipore), and washed many times with the urea buffer to remove bound nucleotides and remaining substrates. Concentrated unfolded proteins were refolded in HMK buffer (20 mm Hepes, pH 7.4, 100 mm KCl, 10 mm MgCl_2_), washed again in the concentrators to remove remaining urea, flash-frozen in liquid nitrogen, and stored at −80 °C.

### Spin labeling

DnaK contains a Cys residue at position 15. This residue does not become labeled when the labeling reaction proceeds in the presence of ATP (46). After elution from the ATP-affinity column with 5 mm ATP, Cys-containing proteins were labeled with MTSL in a 1:3 ratio (∼40 μm protein) in the dark overnight. After labeling, the proteins were unfolded in 8 m urea as described above and refolded in HMK buffer. Labeled proteins were flash-frozen, lyophilized, and stored at −80 °C. Before the electron spin resonance experiments, lyophilized proteins were reconstituted in D_2_O.

### Trp fluorescence emission

Steady-state fluorescence emission of Trp^102^ of DnaK was measured as described previously (46). Fluorescence of 3–5 μm DnaK variants in 20 mm Hepes, pH 7.4, 100 mm KCl, and 5 mm MgCl_2_ (without and with 100 μm ATP) was collected from 305 to 400 nm upon excitation at 295 nm (5-nm bandwidth at both emission and excitation) in a Varian Cary Eclipse fluorescence spectrophotometer (Agilent Technologies) at 20 °C in a quartz cuvette. After measuring the Trp fluorescence of the DnaK variants in the absence of nucleotides, 1 mm ATP was added into the cuvette, mixed, and incubated for 10 min, and the fluorescence from the “ATP-bound” state was collected. Longer incubation times up to 20 min did not change the spectra significantly (not shown). The data were normalized by setting the maximum fluorescence of the apo-state of the non-mutated protein to 1 and normalizing all curves accordingly.

### Ion-mobility native mass spectrometry

20 μm DnaK was incubated in 20 mm Hepes buffer (pH 7.4, 10 mm KCl, 5 mm MgCl_2_) with 200 μm ATP, 200 μm ADP, and 200 μm p5 peptide (CLLLSAPRR; BioMatik), or with 200 mm ATP and 200 μm peptide p5, for 16 h at 4 °C. Buffer was exchanged to 100 mm ammonium acetate, pH 7.5 (Sigma-Aldrich, catalog no. A2706) by Micro Bio-Spin 6 chromatography columns (Bio-Rad) and/or Vivaspin 6 500-μl concentrators (Vivaproducts, Inc.) as described by Hernández and Robinson (47). Samples in ammonium acetate (estimated concentration ∼6 μm) were kept on ice until used. IMMS measurements were performed within 10–20 min of buffer exchange; longer incubation times (hours) resulted in the appearance of species with long arrival times, which we believe are unfolded. 5-μl samples were loaded into house-pulled 1.0-mm outer diameter/0.78-mm inner diameter capillaries for nanospray electrospray ionization as described previously (47). IMMS experiments were performed in triplicate at each ligand-bound condition; individual results and averages are reported in supplemental Fig. S3.

IMMS spectra were recorded on a modified Synapt G1 (Waters, Manchester, UK) equipped with a 32,000 *m*/*z* quadrupole mass filter, where the traveling-wave ion-mobility cell was replaced by an 18-cm helium-filled drift cell with radial RF ion confinement and a linear voltage gradient that directs ions to the time-of-flight (TOF) mass analyzer (48, 49). Spectra were calibrated with cesium iodide (Sigma-Aldrich) at 100 mg ml^−1^ in water (47). All spectrometer conditions were optimized for maintaining the native state of the protein-protein complexes.

Ion-mobility spectrometer parameters in the ES+ mode: sampling cone: 40.0; extraction cone: 1.0; capillary (kV): 1.7; sampling cone: 40.0; source temperature (°C): 20; desolvation; cone gas flow (liters/h): 40.0; nanoflow gas pressure (bar): 0.1; low mass resolution: 4.0; high mass resolution: 15.0; trap collision energy: 10.0 V; transfer collision energy: 70.0 V; trap gas flow (ml/min): 7.00; IM gas flow (ml/min): 50.00; pusher interval (μs): 256; pusher width (μs): 9; pressure and temperature of the ion-mobility cell were measured directly and recorded for each IM voltage condition. Ion-mobility data were taken at 70, 90, 100, 120, 140, 180, and 200 V. Mass spectra and ion-mobility data were analyzed using both MasLynx and Driftscope software (Waters, Manchester, UK).

IMMS of DnaK under each ligand-bound condition yielded an individual MS spectrum (supplemental Fig. S2) and for each ion. ATDs are reported in supplemental Fig. S1. ATD data were exported to Excel (Microsoft Corp.), and the curves were fit to Gaussian components (see supplemental Fig. S1). Curves for DnaK+ATP were fit to two components, and those for ADP/substrate and ATP/substrate were fit to three components. In addition to seeking mathematical improvement of the sum of the square of the differences between the fit and the experimental data (χ^2^), we used additional criteria to decide the number of components for fits of the ATDs; the observation of the largest number of conformers observed (as clear peaks in the ATD) at all voltage enables us to deduce how many species should be included in the analysis of that sample. For example, the ATDs of the ATP-bound sample never show the presence of more than two species regardless of the accelerating voltage or which charge state is picked for the analysis (illustrated in supplemental Fig. S1*A*). In this case, we therefore fit to two Gaussians. In the case of the samples in the presence of ADP/substrate and ATP/substrate, the ATDs of many ions at many acceleration voltages clearly show the presence of at least three Gaussian components (supplemental Fig. S1, *B* and *C*). Fitting was done in Excel, and data were plotted using Kaleidagraph (Synergy Software).

The maximum value for each fitted component was used to calculate the CCS using the Mason-Schamp equation (50). The CCS values for the individual components were used to convert the ATD distributions into CCS distributions (supplemental Fig. S4).

### Pulse dipolar electron spin resonance experiments

25–50 μm DnaK solution was mixed with 1 mm ATP or ADP with or without peptide substrate (NRLLLTG, from BioMatik) in a 1:20 molar ratio for 10 min at 25 °C. Deuterated proline was added to reach a final concentration of 20% (w/v). Standard four-pulse DEER ESR experiments were performed using a home-built Ku band (17.3-GHz) PDS ESR spectrometer at 60 K (30, 51). A pulse sequence with π/2-π-π pulse widths of 16, 32, and 32 ns, respectively, and a 32-ns π pump pulse was routinely used. The frequency separation between detection and pump pulses was 70 MHz. Typical evolution times were 6–8 μs with signal averaging from 2 to 20 h. The homogeneous background was removed from the raw time domain signals, and the distances were reconstructed from the baseline-corrected and normalized signals using the Tikhonov regulation method and refined by the maximum entropy method as described previously (52, 53). We show in Figs. 2 and 3 the form factors of the DEER time-domain signal for each spin pair. From the time domain data, distance distributions, *P*(*r*), were calculated using the L-curve Tikhonov regularization (27, 52). A 4-h acquisition was typically enough to accurately calculate the distance distribution. However, we extended the acquisition time to ∼8–12 h (up to 20 h in few cases) to achieve high signal/noise ratio for longer records. Each distribution profile was fit to Gaussian components, and the position and weights of the distance components (30) are reported in supplemental Table S3.

The distance profiles and the titration data were analyzed by Origin (OriginLab Inc.) and Excel. The interresidue distances were measured on the high-resolution structures of DnaK (PDB entries 2KHO (6) and 4B9Q (8)) between the αC of the residues replaced by Cys in the constructed mutants (Figs. 2 (*B* and *C*) and 3). Additional discussion of the considerations when measuring longer distances by DEER is included in the supplemental data.

## Author contributions

A. L. L., E. M. C., M. E. B., and L. M. G. conceived and coordinated the study and wrote the paper. A. L. L., E. M. C., M. E. B., and L. M. G. designed the experiments performed in Figs. 2 (*B* and *C*), 3, 4, and 5 and supplemental Fig. S5; A. L. L. performed and analyzed the experiments shown in Figs. 2 (*B* and *C*), 3, 4, and 5 and supplemental Fig. S5; and E. M. C. performed and analyzed the experiments shown in Fig. 2*A* and supplemental Figs. S1, S2, S3, and S4. P. P. B. and J. H. F. helped with the acquisition and the interpretation of data for Figs. 2 (*B* and *C*), 3, 4, and 5 and supplemental Figs. S4 and S5, and N. A. P. and C. V. R. helped with the acquisition and the interpretation of data for Fig. 2A and supplemental Figs. S1–S4. All authors reviewed the results and approved the final version of the manuscript.

## Supplementary Material

Supplemental Data

## Footnotes

3 The abbreviations used are:

NBD: nucleotide-binding domain
SBD: substrate-binding domain
βSBD: SBD β-sheet subdomain
αSBD: SBD α-helical lid subdomain
LC: lid closed
DL: dynamic lid
MTSL: *S*-(1-oxyl-2,2,5,5-tetramethyl-2,5-dihydro-1H-pyrrol-3-yl)methyl methanesulfonothioate
AMP-PNP: 5′-adenylyl-β,γ-imidodiphosphate
IMMS: ion-mobility native mass spectrometry
DEER: double electron-electron resonance
CCS: collision cross-section
PDB: Protein Data Bank
ATD: arrival time distribution.
