# Supplementary material for: Key features of an Hsp70 chaperone allosteric landscape revealed by ion-mobility native mass spectrometry and double electron-electron resonance
Source: J Biol Chem. 2017 Apr 20;292(21):8773–85. doi: 10.1074/jbc.M116.770404 (PMC5448104; doi:10.1074/jbc.M116.770404)
Supplement: Supplemental Data [file supp_292_21_8773__index.html]

Key features of an Hsp70 chaperone allosteric landscape revealed by ion-mobility native mass spectrometry and double electron-electron resonance — Allosteric landscape of an Hsp70 chaperone — Supplemental Data 

# Key features of an Hsp70 chaperone allosteric landscape revealed by ion-mobility native mass spectrometry and double electron-electron resonance

## Supplemental Data

- Supplemental data (.pdf, 5.3 MB) - Supplemental text Five supplemental figures
