# Supplementary material for: Key features of an Hsp70 chaperone allosteric landscape revealed by ion-mobility native mass spectrometry and double electron-electron resonance
Source: J Biol Chem. 2017 Apr 20;292(21):8773–85. doi: 10.1074/jbc.M116.770404 (PMC5448104; doi:10.1074/jbc.M116.770404)
Supplement: Supplemental Data [file 10.1074_M116.770404_jbc.M116.770404-1.pdf]

Supplemental Data for:

Key features of an Hsp70 chaperone allosteric landscape revealed by ion mobility native mass spectrometry and double electron-electron resonance

Alex L. Lai[*a], Eugenia M. Clerico[*b], Mandy E. Blackburn[c], Nisha A. Patel[d], Carol V. Robinson[d], Peter P. Borbat[a], Jack H. Freed[a] and Lila M. Gierasch[b,e]

[*]These authors contributed equally.

From the [a]Department of Chemistry and Chemical Biology, Cornell University, 122 Baker Laboratory, Ithaca, NY 14853-2703, USA, the [b]Department of Biochemistry and Molecular Biology, University of Massachusetts, Amherst, 240 Thatcher Rd, Amherst, MA 01003, USA, the [c]School of Environmental, Physical and Applied Sciences, University of Central Missouri, 116 W. South St, Warrensburg MO 64093, USA, the [d]Physical and Theoretical Chemistry Laboratory, South Parks Road, Oxford OX1 3QZ, UK and the [e]Department of Chemistry, University of Massachusetts, Amherst, 240 Thatcher Rd, Amherst, MA 01003, USA.

Supplemental Data:

Supplemental text

Five supplemental figures with legends

Supplemental references

Supplemental text:

DEER distance limits. The long-distance limit of DEER experiments is approximately 80 Å. However, the accuracy of distance reconstruction is reduced when the distance is greater than 65 Å (1), where the peaks broaden, increasing the uncertainty (2). In a single DEER experiment, the evolution time that is required to resolve two peaks in P(r) is not only dependent on the distance separating the peaks, but also on the position of the peaks. For example, to resolve two peaks centered at 53 and 59 Å (as it is the case for the distances measured for ATP/S and ADP/S-bound DnaK* C52 C410), an evolution time of about 7 μs is required. To resolve two peaks at 46 and 49 Å (as it is the case for the distances measured for ATP/S and ADP/S-bound DnaK* C333 C410), an evolution time of about 7 μs is also required. However, an evolution time of about 13 μs would be required to resolve two peaks at 63 and 69 Å. Our experiments typically used 6-8 μs evolution time, which was sufficient to resolve the peaks of 46 and 49 Å and 53 and 59 Å. In the distance reconstruction step, the accuracy of the center position of a peak is typically within 0.5~1 Å if there is no conflicting peak within ± 5 Å of the peak (as in all our data sets). The reliability is further strengthened by repeating the distance measurements for independent samples where distances are reproducible within ± 1 Å.

Supplemental Figures

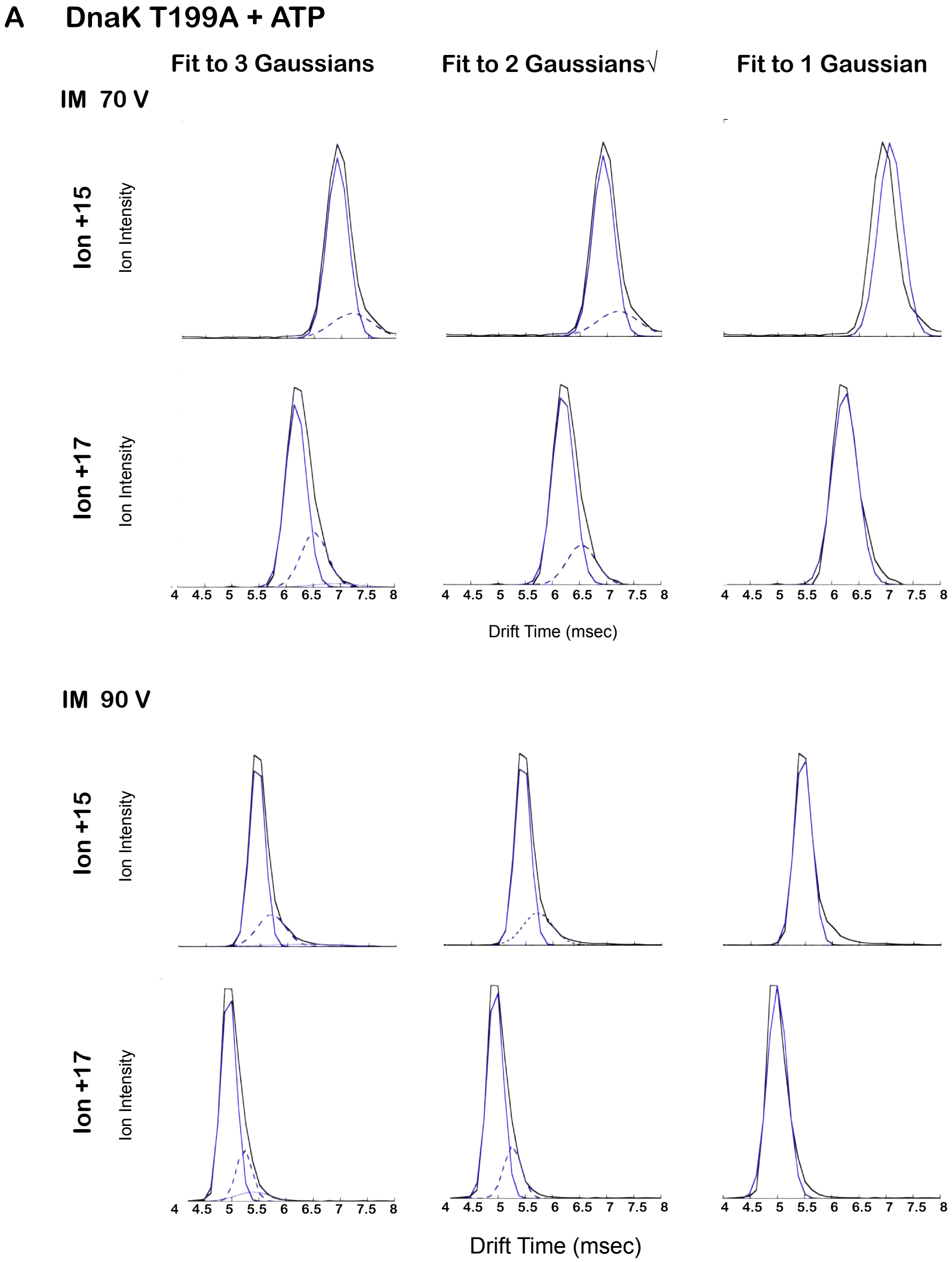

Figure S1. Arrival time distributions (ATDs). Representative set of ATDs for DnaK in the presence of **(A)** ATP, **(B)** ADP/substrate and **(C)** ATP/substrate fit to multiple Gaussian curves. The two most prominent ions in the series at 70 and 90 V (IM voltage) are shown. We used the mathematical improvement of the sum of the square of the differences between the fit and the experimental data (X^2) as one criterion to choose whether to fit some curves to two or three Gaussian components. Additionally, by inspecting the ATDs for all ions at all accelerating IM voltages, we identified the largest number of components displayed by DnaK under defined ligand-bound conditions and deduce how many species should be included in the analysis of that sample. Although these species are not so well resolved for some charge states or acceleration voltages, the fact that they are observed at any defined condition in the IM tube means that (at least) this number of species is present in the ensemble

B DnaK T199A + ADP/Substrate

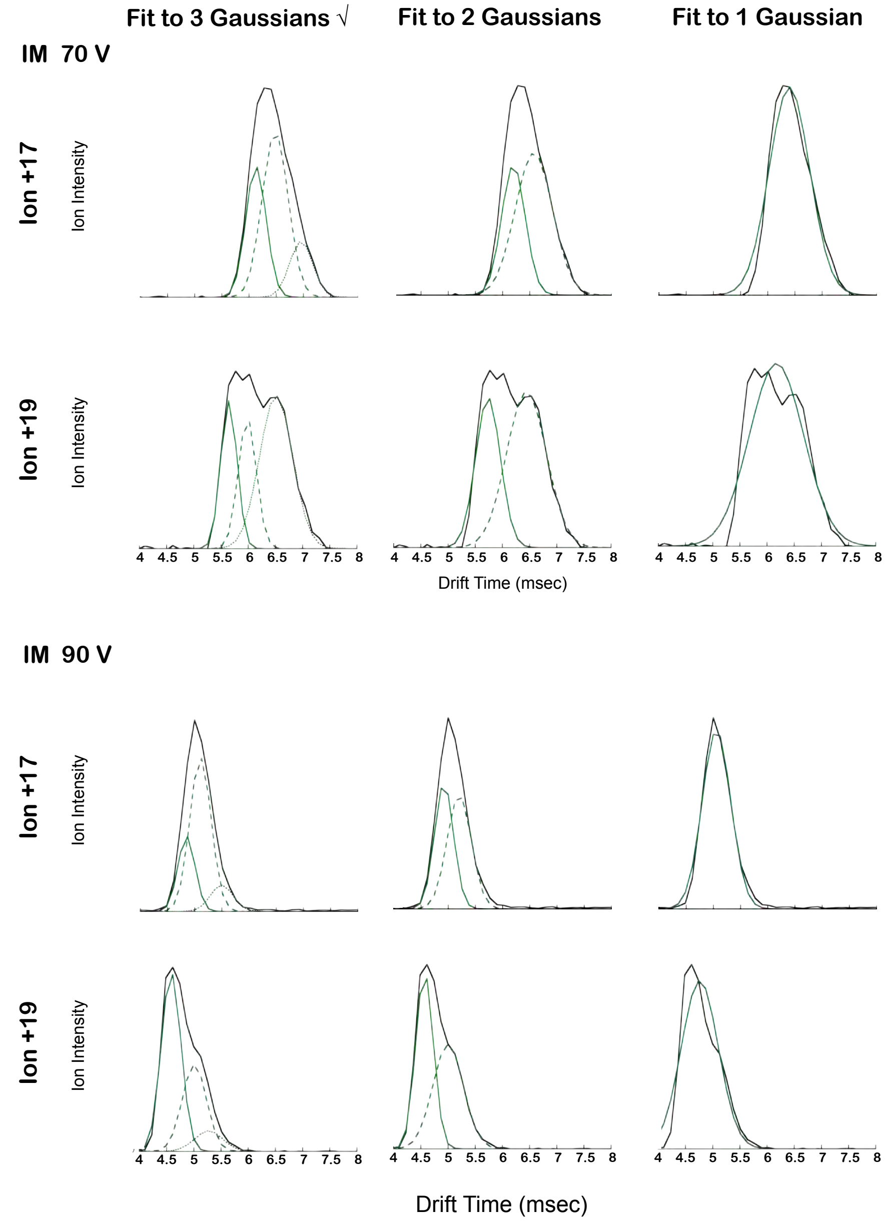

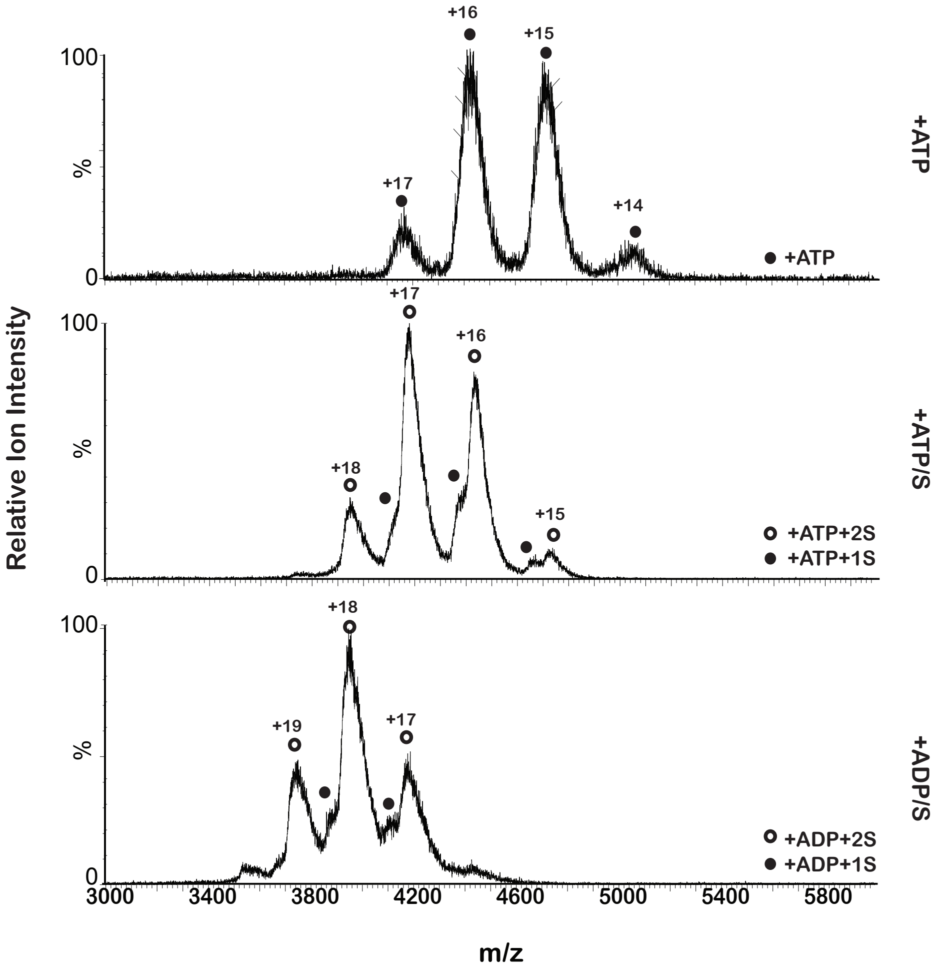

Figure S2 Native mass spectra of DnaK in the presence of ATP, ADP/substrate and ATP/substrate. Shown are m/z distributions corresponding to DnaK bound to the indicated ligands (one representative set of data shown). The masses of all species were calculated using these spectra; but because the error associated with the mass measurement for DnaK +ATP was large, masses were confirmed by native MS in a Q-tof spectrometer (not shown). Note that in the presence of p5, the predominant species is DnaK in complex with two peptides, most likely due to a disulfide linkage between peptides. The presence of two bound peptides causes the same effect on DnaK as one peptide, based on the CCS analysis of the peak corresponding either one or two peptides bound to DnaK +ATP (not shown).

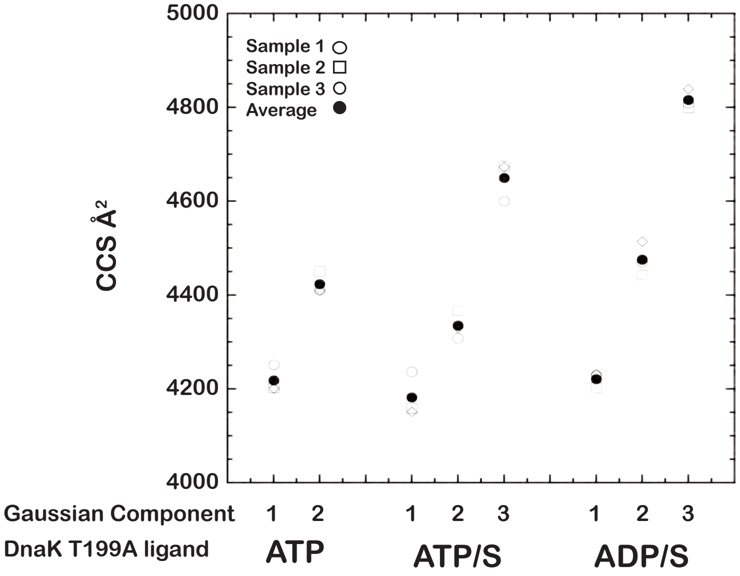

Figure S3. Individual CCS values for the components present for ion +17 for all ligand-bound DnaK. CCS were calculated from the drift times as a function of IM voltage. Individual values for independent experiments are represented in open symbols and the average in filled symbols.

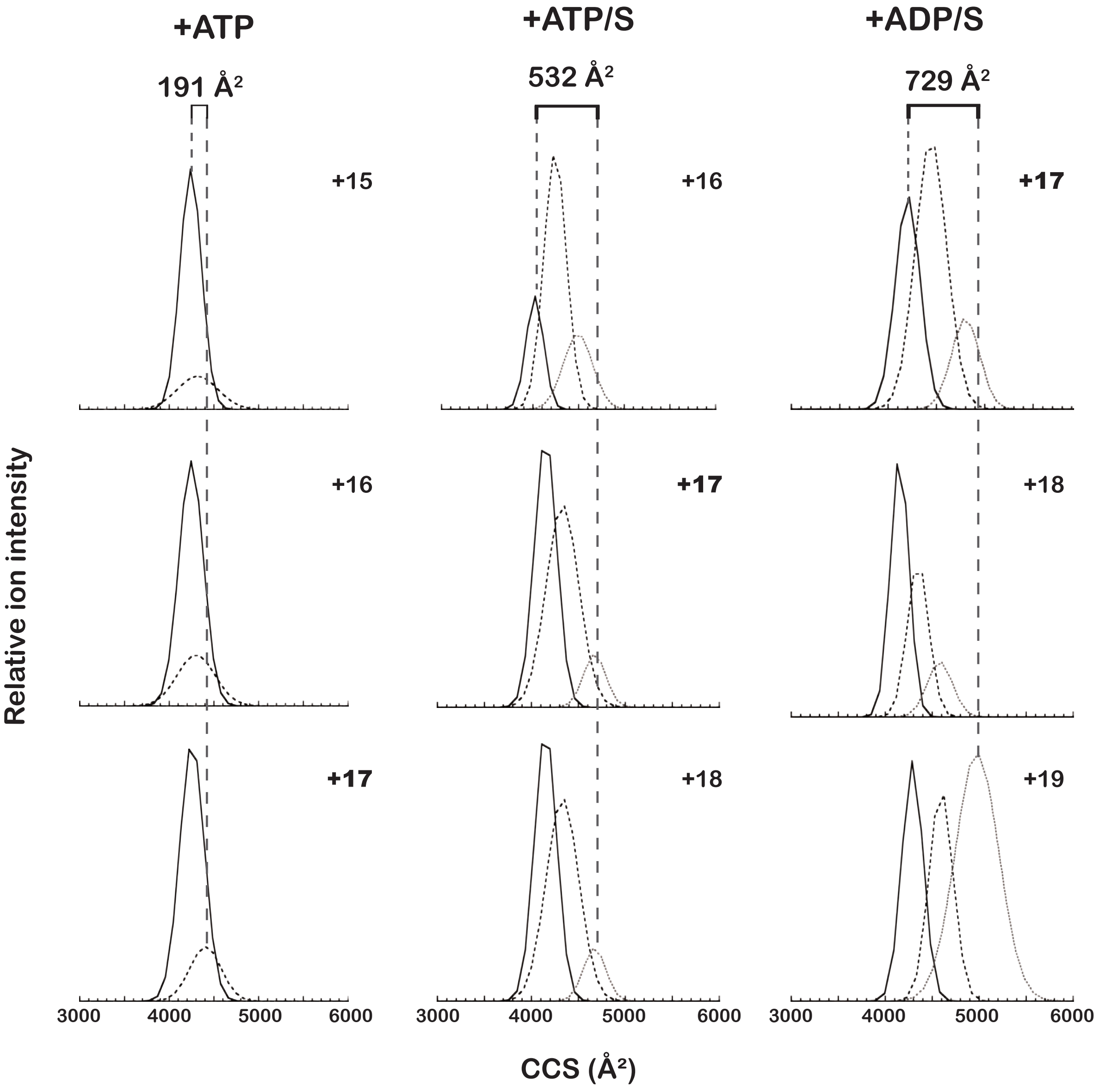

Figure S4. CCS distributions of all DnaK ions in all ligand-bound conditions. CCS values for the components present for each ion were calculated from the drift times as a function of IM voltage. Then the resulting relationship between drift time and CCS values was used to derive the CCS distributions shown for each ion. The different components of the same ion are indicated in solid black, dashed black, dotted black and dotted gray lines. The brackets, dashed gray lines and numbers (CCS in $Å^2$) indicate the breadth of the CCS range spanned by all the ions populated by the protein in a ligand-bound state. This range was calculated as the difference (in $Å^2$) between the CCS of the component with the smallest CCS and the component with the largest CCS found in the indicated condition.

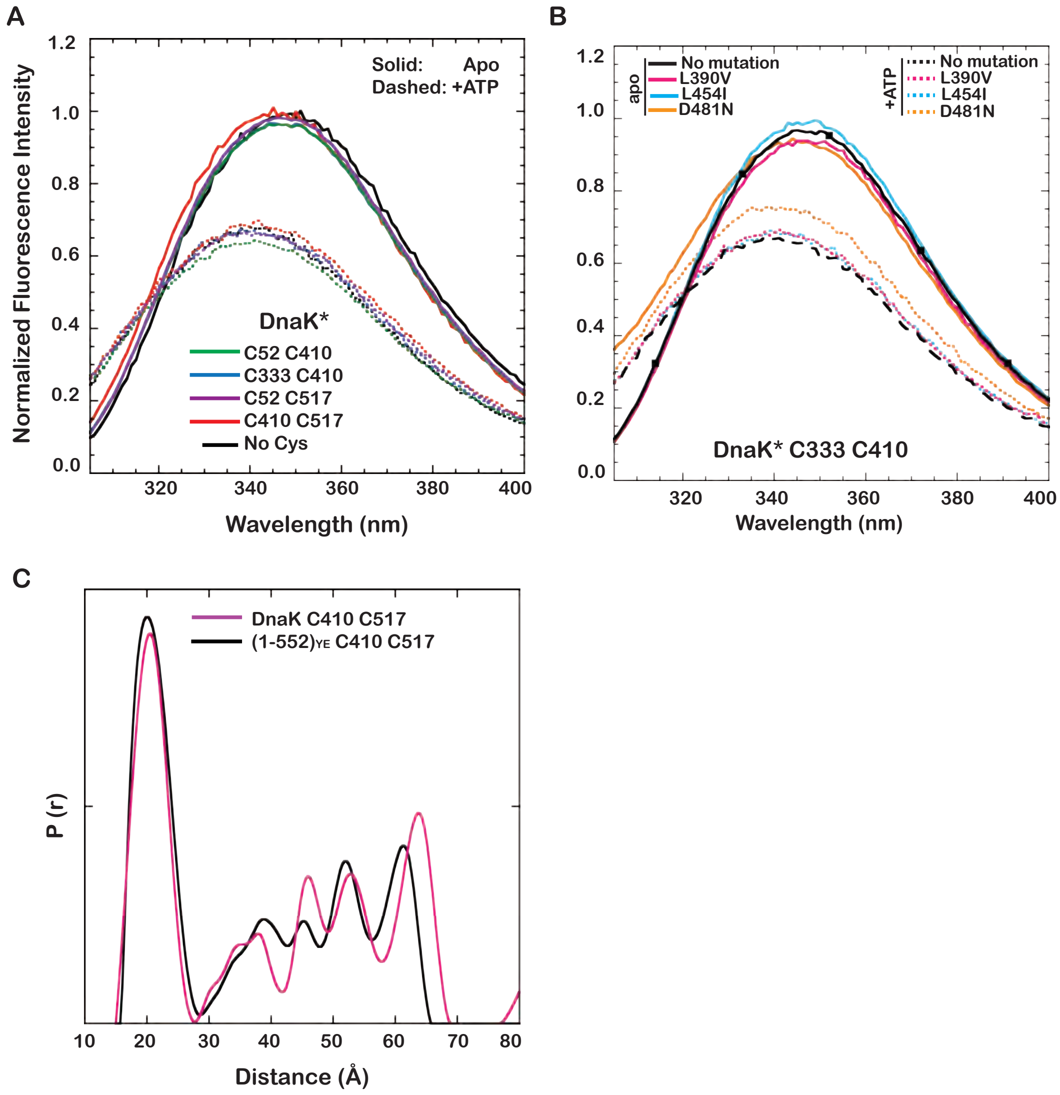

Figure S5: Structural validation of the DnaK mutants used for DEER. A. Allosteric function of the DnaK Cys mutants by Trp fluorescence. DnaK (1-552)$_{YE}$ and its Cys mutants were tested for their ability to adopt the domain-docked state upon ATP-binding by measuring fluorescence emission of Trp 102. Fluorescence data were normalized for concentration and by the fluorescence of the apo-state for each variant. The quenching and the blue shift of the maximum emission that occurs upon ATP binding is the same for all tested DnaK variants. **B.** Allosteric function of the DnaK domain-interface mutants (corresponding to Figure 5 of the main text) by Trp fluorescence as described. **C.** Comparison between full length and (1-552)$_{YE}$ DnaK (both C410 C517) by DEER. Inter-spin distance distributions were compared for full-length DnaK C410 C517 (i.e., 1-641) and DnaK* C410 C517 (truncated) with ATP and NR-bound. The full-length protein has a faster relaxation rate than the truncated variant, possibly due to oligomerization. The phase memory time of DnaK* C410 C517 is 1.14 μs, while that of full-length DnaK* C410 C517 is 0.43 μs. Note that ca. 20 h data acquisition is required to obtain a spectrum for the full-length protein with a similar signal-to-noise ratio as a 2 h run for the truncated counterpart. Thus, we chose to use DnaK* for all DEER measurements.

Supplemental references:

1. Jeschke, G. and Polyhach, Y. (2007) Distance measurements on spin-labelled biomacromolecules by pulsed electron paramagnetic resonance. *Phys. Chem. Chem. Phys.*, **9**, 1895-1910.

2. Borbat, P. P. and Freed, J. H. (2013) Pulse dipolar electron spin resonance: distance measurements, structural information from spin-labels and intrinsic paramagnetic centres, in *The Biosciences*, Volume 152 of the series Structure and Bonding, Springer-Verlag, Berlin Heidelberg; pp. 1-82)
